# Supplementary material for: SeqOthello: querying RNA-seq experiments at scale
Source: Genome Biol. 2018 Oct 19;19:167. doi: 10.1186/s13059-018-1535-9 (PMC6194578; doi:10.1186/s13059-018-1535-9)
Supplement: Supplementary file 1 — Figure S1. The histograms of k-mer occurrence frequencies in two human RNA-Seq datasets. (PDF 154 kb) [file 13059_2018_1535_MOESM1_ESM.pdf]

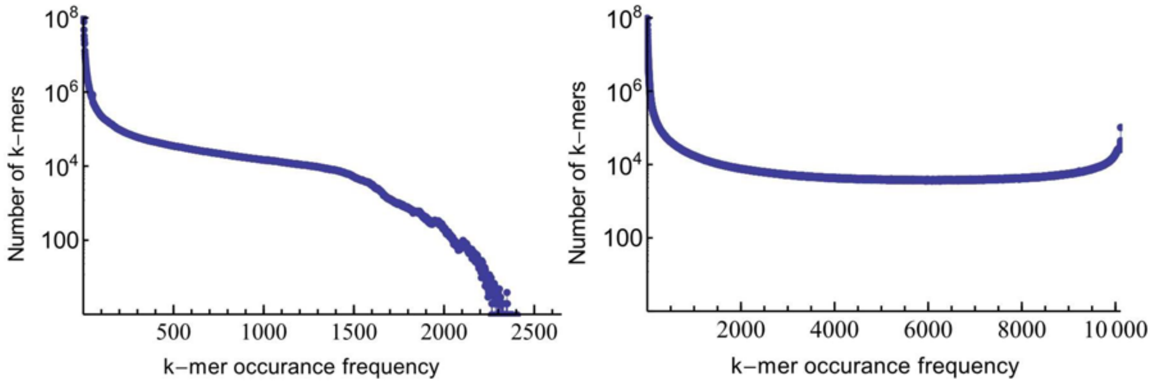

a) 2,652 RNA-seq Experiments from SRA **b)** 10,113 TCGA Pan-Cancer RNA-seq Experiments.

Fig S1. The histograms of k-mer occurrence frequencies in two human RNA-Seq datasets. A k-mer's occurrence frequency is the number of samples containing the k-mer. The difference in the high frequency k-mers between the two datasets suggests less homogeneity in RNAseq experiments downloaded from SRA than these generated by TCGA. a) The k-mer occurrence histogram across 2652 RNA-seq experiments of human blood, breast and brain tissues from the SRA. b) The k-mer occurrence histogram across 10,113 TCGA Pan-Cancer RNA-seq experiments.
