# Supplementary material for: SeqOthello: querying RNA-seq experiments at scale
Source: Genome Biol. 2018 Oct 19;19:167. doi: 10.1186/s13059-018-1535-9 (PMC6194578; doi:10.1186/s13059-018-1535-9)
Supplement: Supplementary file 3 — Table S1 Performance comparison on index construction. (PDF 13 kb) [file 13059_2018_1535_MOESM3_ESM.pdf]

Tools	SeqOthello	SBT	SBT-AS	SSBT
k-mer preparation (**days)**	**3.4**	4.1	4.3	4.8
Index building (**hours**)	**1.9**	39.5	10.2	46.6
Peak memory (GB)	**14.1**	23.4	39.1	5.6
Intermediate disk space (TB)	**0.9**	1.4	3.7	1.9
Final index size (GB)	**20.8**	185.5	168.5	30.8

Table S1: Performance comparison on construction. We compared the time, memory and space usage to construct the SBT[10], SSBT[11] and SBT-AS[12] trees with SeqOthello using the 2,652 SRA RNA-seq samples. The *k*-mer preparation step converts each individual sequencing experiment to the binary format using Jellyfish-based functions with 16 threads. In order to alleviate noise from sequencing errors, *k*-mers having a frequency lower than a specific threshold were removed from the experiment[10] (see Supp table 2). The index step follows the *k*-mer prep step. Unless mentioned otherwise, the comparisons were tested using a single thread. SeqOthello reduces the index construction time by 81% comparing to SBT-AS and the final index size by 32% comparing to the smallest SSBT index.
