# Supplementary material for: SeqOthello: querying RNA-seq experiments at scale
Source: Genome Biol. 2018 Oct 19;19:167. doi: 10.1186/s13059-018-1535-9 (PMC6194578; doi:10.1186/s13059-018-1535-9)
Supplement: Supplementary file 4 — Table S2. Performance comparison on small batch query. (PDF 55 kb) [file 13059_2018_1535_MOESM4_ESM.pdf]

	Θ	max filters	Memory (GB)	Time (min)
SeqOthello-online	-	-	27.3	**1.5**
SeqOthello	-	-	6.1	**4.6**
SBT	.7	1	0.5	78.4
	.8	1	0.5	64.7
	.9	1	0.5	46.9
	Total	1	0.5	190.0
	.9	10,000	175.1	48.9
SBT-AS	.7	1	0.8	14.5
	.8	1	0.8	13.0
	.9	1	0.8	12.5
	Total	1	0.8	40.0
	.9	10,000	131.4	11.6
SSBT	0.7	1	0.2	100.2
	0.8	1	0.2	82.7
	0.9	1	0.2	58.6
	Total	1	0.2	241.5
	0.9	10,000	28.1	55.6

Table S2: Performance comparison on small batch query. We evaluated the memory and time cost for SBT, SBT-AS, SSBT and SeqOthello on querying 10 sets of 1000 transcripts in the 2,652 experiments. We first estimated the expression profiles of the 198,074 GenCodeV25 transcripts for a randomly selected 200 experiments using Sailfish. We then randomlysampled 10 sets of 1000 transcripts that are expressed above 100 TPM (transcripts per million) in at least one of the experiments to build the small batch query sets. This is to make sure the query sets are non-trival. We benchmarked the query performance of SBT, SBT-AS and SSBT using Θ = 0.7, 0.8 and 0.9 with the max-filters setting to 1 and Θ = 0.9 with the max-filters setting to 10,000. The max-filters parameter defines the maximum number of filters loaded in the memory for all SBT-based algorithms. Setting this parameter to 10,000 will make sure the entire bloom filter tree staying in the memory without unnecessary eviction. SeqOthello does not require any parameter for sequence query.
