# Supplementary material for: SeqOthello: querying RNA-seq experiments at scale
Source: Genome Biol. 2018 Oct 19;19:167. doi: 10.1186/s13059-018-1535-9 (PMC6194578; doi:10.1186/s13059-018-1535-9)
Supplement: Supplementary file 5 — Figure S2. A Venn diagram showing the accuracy in querying human transcriptomic k-mers totaling 120,044,842 from experiment SRR925711. (PDF 143 kb) [file 13059_2018_1535_MOESM5_ESM.pdf]

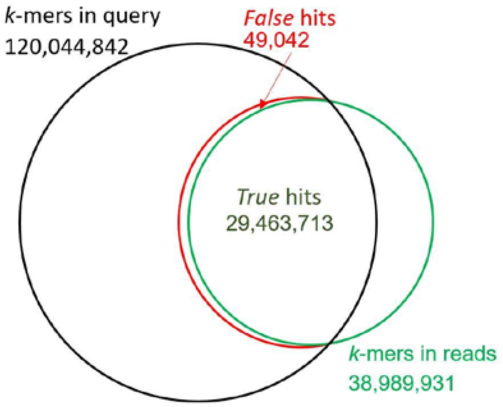

Fig S2. A Venn-Diagram showing the accuracy in querying human transcriptomic k-mers totaling 120,044,842 from experiment SRR925711. There are 120 million transcriptomic k-mers used for the SeqOthello query (the dark circle) and within the particular experiment SRR925711, there exists 39 million k -mers (the green circle). SeqOthello returned a total of 29.5 million hits in the experiment, among which only less than 0.2% is false. This is comparable to error rates found in nextgeneration RNA-seq data. Similar exercises were repeated on 150 experiments to obtain a representative distribution of false positive rate in k-mer query. (Figure 3)
