# Supplementary material for: SeqOthello: querying RNA-seq experiments at scale
Source: Genome Biol. 2018 Oct 19;19:167. doi: 10.1186/s13059-018-1535-9 (PMC6194578; doi:10.1186/s13059-018-1535-9)
Supplement: Supplementary file 7 — Figure S3. An illustration of fusion junction sequence constructed for fusion query using SeqOthello. (PDF 107 kb) [file 13059_2018_1535_MOESM7_ESM.pdf]

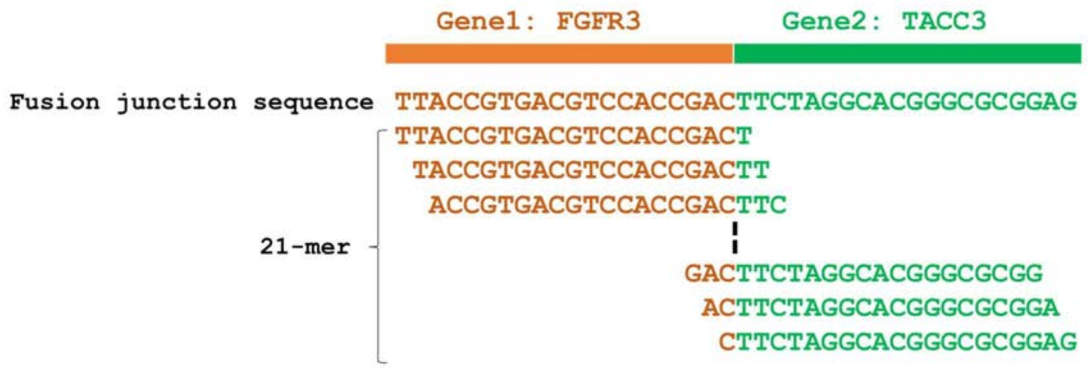

Fig S3. An illustration of fusion junction sequence constructed for fusion query using SeqOthello. Each fusion junction sequence consists of 20 bases from donor exon in one gene and 20 bases from acceptor exon in the other gene. Each 21-mer within this 40-base sequence spans the fusion junction. The query of a fusion sequence using SeqOthello may return a maximum of 20 k-mer hits for each RNA-seq Experiments indexed by SeqOthello.
