# Supplementary material for: SeqOthello: querying RNA-seq experiments at scale
Source: Genome Biol. 2018 Oct 19;19:167. doi: 10.1186/s13059-018-1535-9 (PMC6194578; doi:10.1186/s13059-018-1535-9)
Supplement: Supplementary file 8 — Review history. (DOCX 166 kb) [file 13059_2018_1535_MOESM8_ESM.docx]

Review History

**First round of review**

**Reviewer 1**

**Are the methods appropriate to the aims of the study, are they well described, and are necessary controls included?**
see below

**Are the conclusions adequately supported by the data shown?**

see below

**Are sufficient details provided to allow replication and comparison with related analyses that may have been performed?**

see below

**Does the method perform better than existing methods (as demonstrated by direct comparison with available methods)?**

Yes

**Is the method likely to be of broad utility? Is any software component easy to install and use?**

See below.

**Is the paper of broad interest to others in the field, or of outstanding interest to a broad audience of biologists?**

Yes: I think so. This is a fast moving field and this seems like a pretty significant advance.

Comments to author:

In SeqOthello, the authors present a novel approach to doing low memory query of many thousands of RNA sequencing experiments. The approach is interesting, the results are good, and the software is usable, but we do have many comments that should be addressed before it is considered for publication.

Items:

1. Page 2, line 49: SRA-Blast works with data sets that have long sequence reads. It does not work with short reads, according to the NCBI. (https://www.ncbi.nlm.nih.gov/books/NBK56920/) 
2. Page 3: The introduction section; the review of tools used in large-scale sequence search is missing one important citation for large-scale sequence RNA-seq search (Pandey et al 2017)
3. Page 3, line 34: "close to 300 novel fusions". Why approximate?
4. Page 3, line 35: "The entire survey …..". This sentence is misleading because it excludes the computational resources required for indexing.
5. Figure 1b: I could not generate a similar k-mer hit map using the SeqOthello manual on github. The k-mer hit map generated by SeqOthello has a different design. It is produced for single query sequences but each row reports the presence/absence of each kmer in all experiments
6. Page 4, line 47: The time reported by the authors for creating the index does not consider kmer counting, which is a huge burden. The authors need to add a sentence to describe the resources required for this.
7. Supplementary table 1 does a good job comparing different software packages but I have some comments 
a. Why do different packages behave differently for the step of k-mer preparation if all of them are using Jellyfish to get kmer counts?
b. According to the authors in page 6, the preparation of -mers averages 4 minutes per sample, which is ~7.4 days. This is much more than that shown in the table. 
8. Supplementary table 2: The size of fastq.gz is influenced by the header format of the reads and the degree of compression. Thresholds should be dependent on more accurate parameters like the no of unique k-mers or the sequence content (no of reads * read length). 
9. Page 4, line 48-51: The text describes the index compression features but you refer to supplementary table 3, which describes the memory and time usage of the large batch query.
10. Page 5, line 4-6 and Figure 2: "While SSBT is extremely memory frugal, it is …". The text agrees with the expected SSBT design but does not match figure 2, which shows SSBT consuming more memory than SBT. 
11. Page 5, line 20: In the small query experiment, the query is executed in 0.09 seconds per transcript which is much slower than the rate of execution achieved in the large batch query using the exact same index (35.7 * 60 / 198,093 = ~ 0.01 second).
12. Figure 4: What is the rate of change in mu and theta?
13. Page 7, line 43 and page 17, line 35: The comparison against software packages used for finding fusion transcripts has a major flaw. These packages detect unknown fusions, which is beyond the ability and scope of SeqOthello. Also the related sentence in the abstract is somewhat misleading. SeqOthello did not detect novel fusions but it was able to find known fusions in more tumor samples missed by the original analysis. 
14. Page 7, line 47: "SeqOthello queries on individual sequences as well as their constituent -mers for their presence and absence in each experiment". SeqOthello assesses the presence and absence of kmers but with respect to whole query sequences, it measures the total number of kmer hits. 
15. Page 8, line 26: Authors define Othello as a minimal perfect hashing algorithm. Also they describe it as a collision-free structure in page 4, line 16. However the description of Othello construction in the MetaOthello paper says that cycles can happen during graph construction. As a structure using minimal perfect hashing, how can a cycle in the graph ever happen if each slot in either bitmaps should only be used by one kmer!?! If there is an explanation for having these cycles in the graph, it was solved by ignoring those few kmers from the data structure (according to the MetaOthello paper). Are you using the same approach here? If yes, doesn't this mean that you can't guarantee 100% recall rate as stated in page 5, line 43? 
16. Page 9, line 24-37: Lemma 1 and its proof are missed up. This is the probability of (') = t not x. And ax here would be the fraction of elements has value x and so on. Please revise against the MetaOthello manuscript, section 2.2.3
17. Page 10, line 19: Authors define x as the probability that an alien -mer falls into a class ∈ - . This means the probability to fall in an alien category. Is not this the same definition of 0? Should not this be Alien?
18. Page 11, line39: "it is used only when other options do not work". How can the second option not work? 
19. Page 12, line 28: Authors are using 10 million k-mers, which is an experiment specific number. This should be a generic percentage, shouldn't it? 
20. Page 13, line 20: I could not find options in the software to update a previously made index.
21. Table3: If we run an alien query against n experiments, we can say: 
+ Σne=1 (e) = 1
This does not hold true for the numbers in table 3.

Other edits:
1. Page 6, line 22: "The database documented of 11,658 unique ..". Please check the sentence structure. 
2. Page 9, line 39: the definition of 0 should be in the lemma line not in the proof .

**Reviewer 2:**

**Are the methods appropriate to the aims of the study, are they well described, and are necessary controls included?**
yes

**Are the conclusions adequately supported by the data shown?**

yes

**Are sufficient details provided to allow replication and comparison with related analyses that may have been performed?**

yes

**Does the method perform better than existing methods (as demonstrated by direct comparison with available methods)?**

Yes

**Is the method likely to be of broad utility? Is any software component easy to install and use?**

yes

**Is the paper of broad interest to others in the field, or of outstanding interest to a broad audience of biologists?**

Yes: Current NGS databases (DNA or RNA-seq) are mostly dead material for biologists since they cannot be queried for arbitrary sequence due the computing power required. The type of software introduced here by Yu et al. is going to make this data queryable again, which is quite important (think about Blast for Genbank).

Comments to author:

This manuscript presents SeqOthello, a method for fast transcript detection in large RNA-seq databases. SeqOthello is based on the Othello minimum perfect hash (MPH) structure introduced by the same authors. For each transcript searched, SeqOthello splits the transcript into its k-mer constituents and uses the MPH to retrieve each k-mer's occurence map in all sequence libraries. The main technical advances here are (i) a data structure for storing occurence maps that is optimized for size using an adaptative encoding, and (ii) a way of estimating background k-mer hits and compute how many k-mer hits in a library are needed to call a transcript hit. Performances are evaluated against leading Sequence Bloom Tree (SBT) methods, using a subset of SRA or the full TCGA dataset. As a proof of principle, the method is applied to the identification of fusion transcripts in TCGA.

The huge growth in RNA-seq data calls for novel, efficient query methods. Indeed, alignment-based tools (Blast, Bowtie) are simply too slow for full-scale queries, except in the hand of specialized laboratories. New SBT and MPH-based methods permit full scale search on a regular computer, which is important for the larger community. Here Yu et al. propose a MPH-based tool which is 2 orders of magnitude faster in query times and substantially smaller in storage than concurrent SBT-based systems. This improvement may eventually lead to web based tools capable of opening the corpus of public NGS data to live queries. This means SeqOthello is a significant addition to the software toolbox for NGS data analysis. The C++ code is distributed via Github under an open source license and is properly documented. 

Another point I found interesting in this manuscript is showing that a k-mer index that is not read-aware enables relatively accurate retrieval of fusion transcripts. This task is often thought to require analysis of full reads or pairs of reads. The authors thus significantly move the line on the question. 

Several points still need to be addressed, which are listed below in order of importance. 

While the "delta-98" percentile used here for estimation of background noise is an improvement on the seemingly more arbitrary theta parameter used in SBT, it is not hassle-free either. 98% may be well suited for seeking fusions in the TCGA database, but this value would make no sense when looking for more common transcripts. The authors should acknowledge that this parameter is no "one size fits all" and should be adapted on a case-by-case basis (and possibly suggest how it could be adapted).

Several sentences and figures refer to "novel fusion occurence", which is ambiguous as it suggests SeqOthello is able to discover a truly novel fusion. All searched fusions come from the TCGA gene fusion database. This should be made clear by using for instance "novel occurrence of a known fusion" instead.

Comparison with CPU times of fusion detection algorithms is a somewhat unfair since these algorithm are able to detect novel fusions, while SeqOthello isn't.

How does Othello compare to other minimal perfect hashing schemes such as BBHASH ou EMPHF?

Expressing the global structure size of the SeqOthello databases in bits/kmer would facilitate comparison with other data structures

P.6: "Although -mer information is implicitly stored in bloom filters employed in SBT-based algorithms, efficient implementation of -mer retrieval by these algorithms is not yet available. "
> It is not clear how is k-mer retrieval would be more efficient with SeqOthello than with SBT.

Fig 4c is unclear as how it should be interpreted. Is it supposed to be read as a ROC curve, assuming all novel fusion occurrences are false positives? 

P.8: Saying Othello has demonstrated "superb" scalability somewhat lacks objectivity. 

P.2: the way SRA-blast limitations are presented is unclear. I would say the main limitation is that just a few libraries can be queried at once. 

P.17: Kumal et al 2016: this is Kumar et al. (+ add reference).

**Authors’ response to reviewers**

Comments from Reviewer 1:

1. Page 2, line 49: SRA-Blast works with data sets that have long sequence reads. It does not work with short reads, according to the NCBI. (https://www.ncbi.nlm.nih.gov/books/NBK56920/)

*Response: We have revised the introduction part to make sure our description about SRA is consistent*

*with NCBI.*

2. Page 3: The introduction section; the review of tools used in large-scale sequence search is missing

one important citation for large-scale sequence RNA-seq search (Pandey et al 2017)

*Response: We acknowledged Mantis in the introduction section.*

3. Page 3, line 34: "close to 300 novel fusions". Why approximate?

*Response: We have revised to the exact number, which is 270.*

4. Page 3, line 35: "The entire survey …..". This sentence is misleading because it excludes the computational resources required for indexing.

*Response: In response to your comments, we have reported the time required to build the index.*

5. Figure 1b: I could not generate a similar k-mer hit map using the SeqOthello manual on github. The kmer hit map generated by SeqOthello has a different design. It is produced for single query sequences but each row reports the presence/absence of each kmer in all experiments

*Response: It is true that the hit map in Figure 1b is the transposed version of the output of SeqOthello. We have built a script to support the transformation of these two formats to match the output in the manuscript. The script is provided in transpose.sh and is available in the source code repository*.

6. Page 4, line 47: The time reported by the authors for creating the index does not consider kmer counting, which is a huge burden. The authors need to add a sentence to describe the resources required for this.

*Response: We agree and have added the actual time used for k-mer counting in the description on Page 4 under paragraph “SeqOthello outperforms State-of-the-art algorithms”. Detailed performance at every step of construction, including k-mer counting, was included in Supp Table 1.*

7. Supplementary table 1 does a good job comparing different software packages but I have some comments

a. Why do different packages behave differently for the step of k-mer preparation if all of them are using

Jellyfish to get kmer counts?

*Response: The k-mer preparation step in Supplementary Table 1 was implemented differently among the 4 tools to convert each individual FASTQ file into the corresponding formats for index construction.*

*Although the k-mer counting was accomplished by Jellyfish, all of them perform an additional conversion step that cannot be measured separately. In the SeqOthello workflow, we use the command-line Jellyfish tool to generate the k-mer counts text file. The output file is then used as input for the PreProcess function from the SeqOthello toolkit to sort the kmers alphabetically and generate the binary file. The total running time for SeqOthello is the time for Jellyfish k-mer counting plus the k-mer file conversion to binary. Note that the k-mer text file to binary conversion is very fast. However, the SBT, SBT-AS and SSBT count functions use the Jellyfish C++ library for counting the kmers and encoding them directly into a bloom filter as an output. Since the Jellyfish k-mer counting is done on the fly, we can only measure the total running time for the k-mer counting and the bloom filter construction. The implementations for the uncompressed bloom filter in SBT and SSBT also differ, causing discrepancies in running time.*

b. According to the authors in page 6, the preparation of k-mers averages 4 minutes per sample, which is ~7.4 days. This is much more than that shown in the table.

*Response: Note the time reported on page 6 is for TCGA dataset while Supplementary Table 1 is on the performance of SRA datasets instead of TCGA data. Some of the SRA datasets are much smaller than*

*TCGA datasets, thus, it is much faster to convert than TCGA data.*

8. Supplementary table 2: The size of fastq.gz is influenced by the header format of the reads and the degree of compression. Thresholds should be dependent on more accurate parameters like the no of unique k-mers or the sequence content (no of reads * read length).

*Response: We appreciate the suggestion and agree that a cutoff considering the number of unique k-mers or the sequence content might be more accurate than the file size. As the cutoff needs to be set before kmers are extracted, the cutoff using fastq.gz is the best approximation. Additionally, in order to conduct a fair and confident comparison, we adopted the same parameter settings used for SBT to make sure the testing parameters and performance results are consistent with the original SBT manuscript.*

9. Page 4, line 48-51: The text describes the index compression features but you refer to supplementary table 3, which describes the memory and time usage of the large batch query.

*Response: We apologize for the confusion. We included the wrong reference in the manuscript. The correct reference is Supplementary Table 1, and it is currently fixed in the revised manuscript.*

10. Page 5, line 4-6 and Figure 2: "While SSBT is extremely memory frugal, it is …". The text agrees with the expected SSBT design but does not match figure 2, which shows SSBT consuming more memory than SBT.

*Response: We apologize for the confusion as we included an old version of the performance figure.*

*SSBT is indeed memory frugal as we described. It only takes 2.8G memory during search.*

11. Page 5, line 20: In the small query experiment, the query is executed in 0.09 seconds per transcript which is much slower than the rate of execution achieved in the large batch query using the exact same index (35.7 * 60 / 198,093 = ~ 0.01 second).

*Response: The discrepancy is because a batch query of multiple transcripts is not equivalent to conducting a series of individual single queries of each transcript. During batch query, optimization has been made so that k-mers from many transcripts can be searched together when visiting each node in the SeqOthello index, thus achieving better locality and a faster rate on average. Also, we built the online query mode in a Client/Server structure, so significant time is spent on communication overhead.*

12. Figure 4: What is the rate of change in mu and theta?

*Response: The change of Mu is 1 between two adjacent points and the change of theta is in 0.05. We have added this information in the caption of Figure 4.*

13. Page 7, line 43 and page 17, line 35: The comparison against software packages used for finding fusion transcripts has a major flaw. These packages detect unknown fusions, which is beyond the ability and scope of SeqOthello. Also the related sentence in the abstract is somewhat misleading. SeqOthello did not detect novel fusions but it was able to find known fusions in more tumor samples missed by the original analysis.

*Response: We appreciate the comments and we agree with the reviewer that the comparison between*

*SeqOthello and traditional fusion detection algorithms is not apples to apples as traditional fusion detection algorithms are able to detect novel fusions. Thus, we have added the estimated performance of another hypothetical approach where an aligner, such as STAR or bowtie, is used for targeted sequence search through alignment. The alignment onto the fusion transcript sequence indicates the presence of the transcripts. The total time for STAR using 16 cores is estimated at 1,294 computational hours given 660 billion reads (estimation based on randomly selected 300 samples) in TCGA dataset. Please note that this is not a common practice but is probably a better comparison. The advantage of SeqOthello is that it supports instant query of sequences while both STAR and fusion detection algorithms require reanalyzing individual sequence files per query, quickly adding up the computational cost as well storage and network cost in the case of large sequencing dataset.*

14. Page 7, line 47: "SeqOthello queries on individual sequences as well as their constituent -mers for their presence and absence in each experiment". SeqOthello assesses the presence and absence of kmers but with respect to whole query sequences, it measures the total number of kmer hits.

*Response: We agree with the reviewer that this sentence is confusing. We revise it as follows:*

*“SeqOthello can be parameterized to report either the number of 𝑘-mer hits of a query sequence or the presence/absence information of each constituent 𝑘-mer of the query in individual samples.”*

*With respect to the SeqOthello package, a user may specify the output format with the command line switch “--detail”. When the switch is on, the SeqOthello query program reports the detailed presence/absence for all 𝑘-mers. When the switch is off, it reports the total number of 𝑘-mer hits.*

15. Page 8, line 26: Authors define Othello as a minimal perfect hashing algorithm. Also they describe it as a collision-free structure in page 4, line 16. However the description of Othello construction in the MetaOthello paper says that cycles can happen during graph construction. As a structure using minimal perfect hashing, how can a cycle in the graph ever happen if each slot in either bitmaps should only be used by one kmer!?! If there is an explanation for having these cycles in the graph, it was solved by ignoring those few kmers from the data structure (according to the MetaOthello paper). Are you using the same approach here? If yes, doesn't this mean that you can't guarantee 100% recall rate as stated in page

5, line 43?

*Response: We appreciate your comments. Othello can be collision-free if the two hashing functions adopted can generate an acyclic graph given the set of the keys. The strict form of Othello will try different pairs of hashing functions until it is acyclic. However, a pair of hashing functions can be found in one try or many tries, depending on the set of keys. The metaOthello data structure involves 6 billion keys, the chance of having 1 key resulting in a cycle is very high given any pair of hashing functions. In the case where a perfect pair of hashing function cannot be found at a few tries, our heuristic allows the removal of some keys (no more than 100) which significantly decreases the construction time without affecting the accuracy as the sequencing datasets and k-mer query are highly redundant.*

*SeqOthello adopts similar principle during the root-level Othello construction. Here, the recall rate has a lower bound of 99.999995%. As this is not exactly 100%, we have removed the claim here.*

16. Page 9, line 24-37: Lemma 1 and its proof are missed up. This is the probability of (') = t not x. And ax here would be the fraction of elements has value x and so on. Please revise against the MetaOthello

manuscript, section 2.2.

*Response: In Metaothello, we use (𝑡) to denote Probability of 𝜏(𝑠’) = 𝑡. In this paper we use 𝑝􀯫 to denote the probability of (𝑠’) = 𝑥. We apologize for the inconvenience but please note that there are more notations presented in this paper, and we believe using notation 𝑝x is better for the content of this paper.*

*We fixed the errors in the equation.*

17. Page 10, line 19: Authors define x as the probability that an alien k -mer falls into a class ∈ - . This means the probability to fall in an alien category. Is not this the same definition of 0? Should not this be

Alien?

*Response: A query with 𝜏 return any value in the range 𝐶 = {0,1, … , 2^l^ − 1}, where only the values in*

*𝑉 = {1,2, ⋯ , 𝑣} are associated to the non-alien keys. The remaining values are associated with alien keys.*

*Therefore, a key 𝑠’ is detected to be alien if (𝑠’) = 0 or 𝜏(𝑠’) ∈ {𝑣 + 1, 𝑣 + 2, ⋯ , 2 ^l^ − 1}.*

18. Page 11, line39: "it is used only when other options do not work". How can the second option not

work?

*Response: We agree with the reviewer that describing it as “do not work” is incorrect. We have revised it to the following:*

*“It is only used when value-list encoding, and delta-list encoding cannot generate a shorter encoding result than that by the bitmap approach”.*

19. Page 12, line 28: Authors are using 10 million k-mers, which is an experiment specific number. This

should be a generic percentage, shouldn't it?

*Response: There is a practical consideration in using a fixed number of 𝑘-mers instead of a fraction of 𝑘-mers. The goal of using the sampled 𝑘-mers is to optimize the construction time by going through the entire 𝑘-mer files once. Should a percentage be given, we will need an additional scan of all 𝑘-mers in order to get the total number of 𝑘-mers, which is likely to double the construction time.*

*In practice, we expect the total number of 𝑘-mers to be around a couple of billions or less. Using 10 million k-mers provides a sufficient sampling of at least 0.5% of the entire k-mer set. Additionally, this number can also be customized by using command line parameter “--estimate-limit’. The default value is 10485760.*

20. Page 13, line 20: I could not find options in the software to update a previously made index.

*Response: We provide an example of building a SeqOthello index structure with additional experiments. The user may reuse the temporary ‘group’ files generated during the build process or the original SeqOthello index. Please refer to the ‘An example of adding new experiments to existing SeqOthello.’ section in the manual. https://github.com/LiuBioinfo/SeqOthello/blob/frozen/README.md*

21. Table3: If we run an alien query against n experiments, we can say:

+ Σne=1 (e) = 1

This does not hold true for the numbers in table 3.

*Response: Sorry we were not able to see the math formula in the comment. It seems the formula was not correctly converted to text. What we can see is ‘+ Σne=1 (e) = 1’. We guessed that the reviewer might wonder if the sum of an alien 𝑘-mer queries on all experiments is 1. The response bellows tries to clarify this question. Please let us know if this is not correct.*

*Please note that we should not compute the sum of the probability values for alien k-mer being detected across all experiments. Consider the set of RNA-seq experiments 𝐸. For any alien 𝑘-mer 𝑠’, and an RNAseq experiment 𝑒 ∈ 𝐸, let EVENT_e_ be the event of 𝑠’ being falsely reported as present for experiment 𝑒.*

*These events are not exclusive to each other as a k-mer may be reported to exist in multiple experiments at the same time. According to the rule for probability of non-mutually exclusive events, they are not supposed to add up to 1.*

22. Other edits:

Page 6, line 22: "The database documented of 11,658 unique ..". Please check the sentence structure.

Page 9, line 39: the definition of 0 should be in the lemma line not in the proof.

*Response: This is corrected.*

Reviewer #2:

1. While the "delta-98" percentile used here for estimation of background noise is an improvement on the seemingly more arbitrary theta parameter used in SBT, it is not hassle-free either. 98% may be well suited for seeking fusions in the TCGA database, but this value would make no sense when looking for more common transcripts. The authors should acknowledge that this parameter is no "one size fits all" and should be adapted on a case-by-case basis (and possibly suggest how it could be adapted).

*Response: We agree with the reviewers that this threshold should be determined on a case-by-case basis. We added the acknowledgement on this at the end of the paragraph where delta was described.*

*“Please note that the background noise 𝛿 should be adjusted according to the expected signal to noise ratio in individual applications.”*

2. Several sentences and figures refer to "novel fusion occurence", which is ambiguous as it suggests

SeqOthello is able to discover a truly novel fusion. All searched fusions come from the TCGA gene fusion database. This should be made clear by using for instance "novel occurrence of a known fusion" instead.

*Response: We agree with the reviewer that novel occurrence of a known fusion is a more accurate description of the discovery. Thus we have revised the manuscript at multiple places to reflect the change.*

3. Comparison with CPU times of fusion detection algorithms is a somewhat unfair since these algorithm are able to detect novel fusions, while SeqOthello isn't.

*Response: We agree with the reviewer that the comparison of SeqOthello with traditional fusion detection software is not 100% apples to apples. We have revised with a more straightforward comparison leveraging STAR. Please see the response to comment 13 from Reviewer 1.*

4. How does Othello compare to other minimal perfect hashing schemes such as BBHASH or EMPHF?

*Response: Othello intend to serve as a hashing classifier, not a minimum perfect hashing function. For a list of keys 𝑆 = {𝑠_1_, 𝑠_2_, ⋯ , 𝑠_n_ }, Othello maintains a many-to-one mapping function τ, where each of the key is mapped into one of the m categories, (in our case, one of the buckets or indices). Here, 𝑚 = 2 ^l^, whichmeans the query result of Othello is always an 𝑙-bit integer. In comparison, a Minimal Perfect Hashing Function (MPHF) for set 𝑆 always maintains a one-to-one mapping from keys to a unique value in the range of 0 to |𝑆| − 1.*

*We say Othello is a minimal perfect hashing classifier because the underlying algorithm of Othello is built upon a MPHF algorithm (B. S. Majewski, N. Wormald, G. Havas, and Z. Czech. A Family of Perfect Hashing Methods. Comput. J., Jun 1996). However, Othello is intended to serve as an indexing tool that demonstrates good query performance with small memory size and is more general compared to a MPHF.*

*In response to your comments, we conducted additional experiments and compared the performance of Othello with BBHash. Here, we select 𝑙 = 8, or 𝑙 = 12, or 𝑙 = ⌈log_2_ 𝑛⌉. The case of 𝑙 = ⌈log_2_ 𝑛⌉ mimics the situation in which we use Othello as a MPHF. We compare this MPHF with BBHash and present the result in the following table:*

Table 1: Comparison between Othello and BBHash as minimal perfect hashing.

*We used Othello to build a minimal perfect hashing function classifier, or MPHF, and compared it with*

*BBHash. The evaluation was conducted using the benchmark tool provided by BBHash. For Othello the value length is set to 8, 12, or ⌈log􀬶 𝑛⌉, where 𝑛 is the number of keys. The corresponding number of categories is 256, 4096, or 𝑛. For BBHash we use two different settings, (Gamma =1 and Gamma =20). We compare their latency, construction time and memory efficiency. “Latency” refers to the average time used to complete one query, which is measured using the procedures in BBHash source code. “Build time” is the time to build the mapping. “Bit/key” shows the memory overhead of Othello and BBHash.*

*Othello demonstrated faster query speed with larger build time and memory overhead compared to BBHash. The experiments were conducted on a Linux OS (RHEL) sever with Quad Intel E5-4640 8 core*

*(Sandy Bridge) @ 2.4 GHz processors, 512 GB of 1600 Mhz RAM, and 4 x 1 TB local (internal) NLSAS disk. The source code for such comparison is also available on the GitHub repository of SeqOthello.*

5. Expressing the global structure size of the SeqOthello databases in bits/kmer would facilitate comparison with other data structures

*Response: This is a great idea for pure hashing data structure such as Othello and BBHash. We have provided the bits/kmer information for the comparison of Othello and BBHash in the table above.*

*However, because SeqOthello data structure involves a hierarchical index structure together with k-mer presence information of many samples, bits/kmer may not be appropriate for SeqOthello or SBT.*

6. P.6: "Although -mer information is implicitly stored in bloom filters employed in SBT-basedalgorithms, efficient implementation of -mer retrieval by these algorithms is not yet available. " It is not clear how is k-mer retrieval would be more efficient with SeqOthello than with SBT.

*Response: SeqOthello and SBT use drastically different strategies in storing 𝑘-mer presence/absence information. SBT is a sample-centric approach. It uses one bloom filter to store all 𝑘-mers in one sample. As a result, it has a collection of bloom filters for all samples. To extract one k-mer, it will need to visit all bloom filters in the worst-case scenario. The tree-based strategy of SBT tries to minimize such sequential search by using internal node of the trees to store aggregated 𝑘-mer information in a branch subset of samples. Such strategy becomes quite effective for 𝜃-based query where a sequence search down the tree can be stopped once plenty of 𝑘-mer presence are identified.*

*SeqOthello is a 𝑘-mer centric approach. It stores the bitmap of 𝑘-mer presence/absence information across all samples. One the 𝑘-mer is identified, its absence/presence information in all samples can be retrieved at one memory location. Therefore, SeqOthello can be much faster than SBT in 𝑘-mer retrieval.*

7. Fig 4c is unclear as how it should be interpreted. Is it supposed to be read as a ROC curve, assuming all novel fusion occurrences are false positives?

*Response: Fig 4c is not exactly a ROC curve as we cannot calculate false positive rate and true positive rate due to the lack of ground truth. We agree with the reviewer that it can be read like a ROC curve as it shows the balance between the detection of known occurrences and the detection of novel occurrences as we change corresponding parameters. The assumption is that novel fusion occurrences do exist but not in an excessive amount.*

8. P.8: Saying Othello has demonstrated "superb" scalability somewhat lacks objectivity.

*Response: We agree with the reviewer and have removed this statement.*

9. P.2: the way SRA-blast limitations are presented is unclear. I would say the main limitation is that just a few libraries can be queried at once.

*Response: We agree with the reviewer and this is revised.*

10. P.17: Kumal et al 2016: this is Kumar et al. (+ add reference).

*Response: This is corrected.*

**Second round of review**

**Reviewer 1:**

The authors did a very good job handling all

the comments and the manuscript reads very well.

**Reviewer 2:**

I thank the authors for their in depth answers. I am satisfied with the proposed changes. I suggest the following minor corrections:

The concept of Minimal Perfect Hashing Classifier is not common. It could be introduced and compared to the more common MPH.

I am not sure the new sentence on SRA Blast being limited to long reads is accurate. I have been able to search some Illumina libraries with 50nt-long reads (just try SRX3636512). Perhaps just write: "SRA-Blast is able to search only a limited set of sequencing experiments"

**Authors’ response to reviewers**

We would like to thank reviewer 2 for the suggestions. We have changed the description regarding SRA-blast in the introduction and have added a descriptive comparison between MPHF and Othello in the method section.
